# eHealth Interventions for Anxiety Management Targeting Young Children and Adolescents: Exploratory Review

**DOI:** 10.2196/pediatrics.7248

**Published:** 2018-05-10

**Authors:** Federica Tozzi, Iolie Nicolaidou, Anastasia Galani, Athos Antoniades

**Affiliations:** ^1^ Stremble Ventures Ltd Limassol Cyprus; ^2^ Department of Communication and Internet Studies Cyprus University of Technology Limassol Cyprus

**Keywords:** child, adolescent, anxiety, anxiety disorders, telemedicine, eHealth, mobile applications, review

## Abstract

**Background:**

Advances in technology are progressively more relevant to the clinical practice of psychology and mental health services generally. Studies indicate that technology facilitates the delivery of interventions, such as cognitive behavioral therapy, in the treatment of psychological disorders in adults, such as depression, anxiety, obsessive-compulsive disorder, panic symptoms, and eating disorders. Fewer data exist for computer-based (stand-alone, self-help) and computer-assisted (in combination with face-to-face therapy, or therapist guided) programs for youth.

**Objective:**

Our objective was to summarize and critically review the literature evaluating the acceptability and efficacy of using technology with treatment and prevention programs for anxiety in young children and adolescents. The aim was to improve the understanding of what would be critical for future development of effective technology-based interventions.

**Methods:**

We conducted an exploratory review of the literature through searches in 3 scientific electronic databases (PsycINFO, ScienceDirect, and PubMed). We used keywords in various combinations: child or children, adolescent, preschool children, anxiety, intervention or treatment or program, smartphone applications or apps, online or Web-based tool, computer-based tool, internet-based tool, serious games, cognitive behavioral therapy or CBT, biofeedback, and mindfulness. For inclusion, articles had to (1) employ a technological therapeutic tool with or without the guidance of a therapist; (2) be specific for treatment or prevention of anxiety disorders in children or adolescents; (3) be published between 2000 and 2018; and (4) be published in English and in scientific peer-reviewed journals.

**Results:**

We identified and examined 197 articles deemed to be relevant. Of these, we excluded 164 because they did not satisfy 1 or more of the requirements. The final review comprised 19 programs. Published studies demonstrated promising results in reducing anxiety, especially relative to the application of cognitive behavioral therapy with technology. For those programs demonstrating efficacy, no difference was noted when compared with traditional interventions. Other approaches have been applied to technology-based interventions with inconclusive results. Most programs were developed to be used concurrently with traditional treatments and lacked long-term evaluation. Very little has been done in terms of prevention interventions.

**Conclusions:**

Future development of eHealth programs for anxiety management in children will have to address several unmet needs and overcome key challenges. Although developmental stages may limit the applicability to preschool children, prevention should start in early ages. Self-help formats and personalization are highly relevant for large-scale dissemination. Automated data collection should be built in for program evaluation and effectiveness assessment. And finally, a strategy to stimulate motivation to play and maintain high adherence should be carefully considered.

## Introduction

Anxiety disorders are among the most common diagnosed mental health problems in children and adolescents [1]. A lifetime prevalence as high as 30% prior to 18 years of age has been reported in American adolescents from the general population, with a median age of onset of 6 years [2,3]. Furthermore, the prevalence of subclinical anxiety has been estimated at a much larger proportion, reaching 40% in children. The most frequent diagnoses are separation anxiety disorder, specific and social phobias, generalized anxiety disorders, agoraphobia, and panic disorder, with these last 3 tending to have a higher incidence in adolescence than in childhood [2].

Cognitive behavioral therapy (CBT) has been demonstrated to be effective in treating children and adolescents with anxiety disorders [4,5]. Other approaches, such as biofeedback, mindfulness, and other relaxation techniques, have also been commonly used [5,6] having shown some efficacy [7-9]. However, the vast majority of children do not receive treatment: a national survey in the United States estimated that up to 80% of youth with a diagnosable anxiety disorder never received specialized mental health care [10].

Such a low access to treatment may have several origins: cost, especially relevant for families and countries with socioeconomic difficulties; geographic location as an impediment to physically accessing care; shortages of specialized providers compared with demand and poor coordination among different services such as schools, primary health care providers, and social services; issues related to stigma associated with receiving mental health services and poor acceptance of treatment, especially among adolescents; and access that is particularly inadequate for vulnerable groups [11-13].

Lack of an early mental health intervention has a significant impact on children’s quality of life and may disrupt their development. Persistently elevated levels of anxiety in children can have an impact on academic performance with school difficulties, and impaired social and emotional functioning [14,15]. Furthermore, the presence of anxiety disorders in young age, as well as subclinical anxiety symptoms, appears to be associated with the risk for the development of anxiety and mood disorders later in life, with a peak in 13- to 15-year-olds [15-17]. It is therefore important to find means to increase the availability of treatment for youth with anxiety.

Recently, there has been broad interest in the use of digital technology to deliver therapies, with the goal of facilitating access to therapy and reducing costs. CBT has been noted to be well suited for remote delivery due to its highly organized content and demonstrated efficacy [18,19], to the point that UK National Institute for Health and Clinical Excellence guidelines have been issued for computerized CBT for depression and anxiety, at least for adults [20].

The use of computer-based health intervention is particularly suited for youth. Nowadays, digital games play an essential role in people’s lives worldwide [21-23], with millions of people from all sociodemographic groups playing digital games in their leisure time [24]. Smartphones are extensively used worldwide [25], and a growing number of health apps for mobile phones and tablets, including mood diaries and mindfulness exercises, are now available [26,27]. Furthermore, serious games can add the element of fun, a component that motivates and enhances learning and behavior change [28].

With this work, we aimed to collect information related to available technology-based programs for anxiety management in young individuals. Our goal was to evaluate existing tools and identify their strengths and weaknesses, with the objective of identifying areas for future research and development. It is important to note that this work built on the results of a recent metareview on eHealth interventions, which included a variety of mental health problems, such as attention-deficit/hyperactivity disorder, autism, depression, psychosis, eating disorders, and posttraumatic stress disorder [29], in addition to anxiety, which was the sole focus of our review. Moreover, previous reviews either targeted adolescents and young adults [29], as opposed to this study, which focused on young children as well, or focused exclusively on CBT-based eHealth interventions for anxiety and depression [30], whereas this study included a variety of approaches that were used for anxiety management, such as biofeedback and mindfulness, in addition to CBT.

## Methods

Because of the small number of published studies (in particular from peer‐reviewed sources) and their heterogeneity, as there were significant differences in the methods and data used, the literature did not lend itself to a more thorough systematic literature review or a meta‐analysis [31]. As it was not possible to apply traditional methods of systematic reviews, such as the Preferred Reporting Items for Systematic Reviews and Meta-Analyses statement, we undertook an exploratory literature review that built on the approach set out by Arksey and O’Malley [32].

We first conducted a systematic literature search in 3 scientific electronic databases: PsycINFO, ScienceDirect, and PubMed; we subsequently also searched Google Scholar. To retrieve articles, we used keywords in various combinations: child/children, adolescent, preschool children, anxiety, intervention or treatment or program, smartphone applications or apps, online or Web-based tool, computer-based tool, internet-based tool, serious games, CBT, biofeedback, and mindfulness. We combined keywords using Boolean operators, for example, “children OR child AND anxiety AND (intervention OR treatment OR program). In addition, we searched a clinical trial register (ClinicalTrials.gov) to detect either ongoing or completed trials that had not been published yet. In the identified articles, we examined references to trace pertinent articles that we might have missed in the search. After retrieving the articles, we removed duplicates and reviewed the remaining titles, then screened the abstracts for potential relevance and carefully read full-text articles relevant to the topic. For inclusion, articles had to (1) employ a technological therapeutic tool with or without the guidance of a therapist; (2) be specific for treatment or prevention, or both, of anxiety disorders in children or adolescents, or both; (3) be published between 2000 and 2018; and (4) be published in English and in scientific peer-reviewed journals. We excluded gray literature and white papers from the review. We excluded programs for obsessive-compulsive disorder and posttraumatic stress disorder because of their low prevalence rates in early childhood and the specificity of both assessment and therapeutic approach. We used no other search limits.

## Results

**Figure 1:**
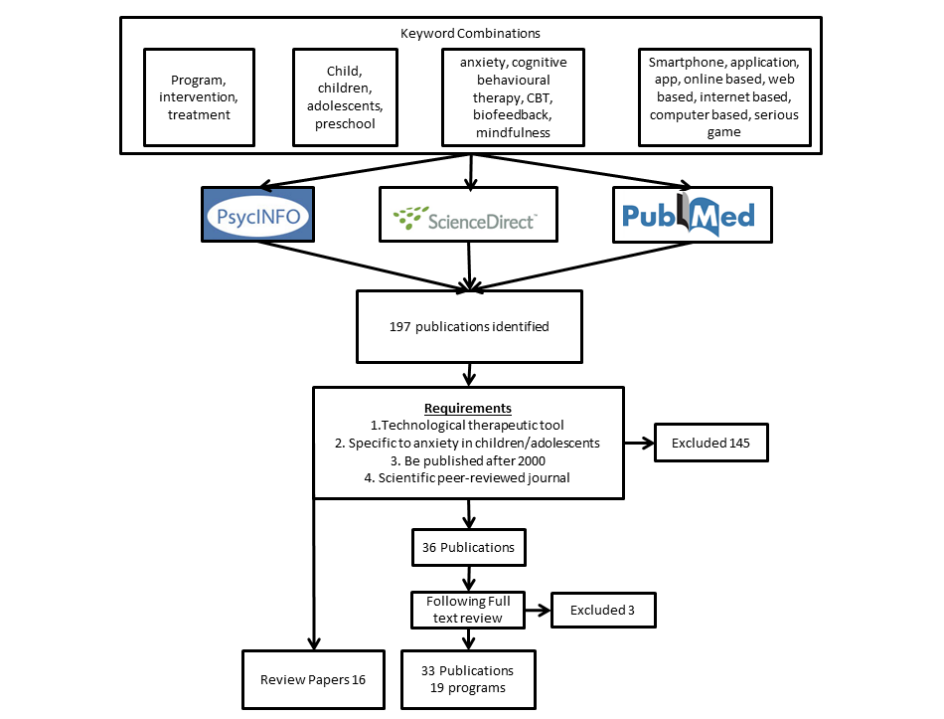
Study flow diagram.

Most programs that we found on the Web were directed at adults or did not specify an age range, with a much smaller number being specifically designed for children. We initially found and examined 197 articles deemed to be relevant. Of these, we excluded 164 because they did not satisfy 1 or more of the requirements outlined above. The final review comprised 19 programs, including technological tools and data for therapeutic treatment or prevention (Figure 1).

To create a taxonomy, we first grouped the programs based on the therapeutic approach they used: most of them fell into two categories, namely CBT and biofeedback. Second, there are 3 age groups that need to be considered when developing a computer-based treatment, reflecting developmental stages: preschool children (2-5 years), children (6-12 years), and adolescents (13-18 years). It is important to note that we use the term *program* to refer to an intervention and the term *tool* to refer to any technology-based components, such as an app for mobile devices, or a CD for desktop or laptop computers. Multimedia Appendix 1 [33] shows a glossary of terms used when evaluating programs.

### Cognitive Behavioral Therapy–Based Programs

CBT has been shown to be highly effective in treating and preventing anxiety disorders among children and adolescents [4,5,34,35] and it is regarded as the first-line choice for this patient population [36].

Our search found 14 technology-based treatments that used CBT as their theoretical framework. Half of them covered the age group of 6 to 12 years. Table 1 [37-61] summarizes the main characteristics of all CBT-based treatments.

**Table 1:** Cognitive behavioral therapy (CBT)–based programs: technology-based tools used in support of traditional CBT therapy programs.

First author, year, reference^a^	Tool name	Target population	Theoretical framework and platform	External support	Automated decision support and data collection	Cost and number of users	Country and language	Structured pilot or trial
Khanna, 2008 [37]; Khanna, 2010 [38]; Storch, 2015 [39]; Crawford, 2013 [40]; University of South Florida, 2016 [41]	Camp Cope-A-Lot (based on Coping Cat)	7-13 y; anxiety disorders	CBT-based CD-ROM-assisted treatment; 12 sessions	Professional and parents; no equipment required	No	US $200 for base package	US; English	Feasibility acceptability study: n=30 (7-12 y); Pilot single group: n=17 (7-13 y) RCT^b^trials: n=49 (7-13 y), N=100 (7-13 y); RCT: n=188 (7-13 y)
Pramana, 2014 [42]; University of Pittsburgh, 2014 [43]	SmartCAT (based on Brief Coping Cat)	7-13 y; anxiety disorders	CBT mobile phone app (Android, iPhone under development) and therapist portal; 8 sessions	Professional; no equipment required	No		US; English	Feasibility study: n=9 (9-14 y); single group: n=40 (9-14 y)
Brezinka, 2014 [44]	Treasure Hunt	9-13 y; anxiety and depression	CBT Web-based computer game with 6 levels; 2.5-dimension Flash with ActionScript (Windows and Mac)	Professional; no equipment required	No	Free access to therapists; >2700 accredited users in 45 countries	English, German, Dutch, and Greek	Nonrandomized, uncontrolled applicability study: N=124 professionals; n=218 children (6-19 y) (through 42 professionals)
Spence, 2006; [45]; March, 2009 [46]; Donovan, 2014 [47]; Spence, 2011 [48]	BRAVE	8-12 y, 13-17 y (+parents); anxiety disorders	Web-based; 12 sessions	Therapist program (minimal contact via phone or email); self-help program; no equipment required	No	Free (available only in Australia)	Australia; English	RCT: n=72 (7-14 y); RCT: n=73 (7-12 y); RCT; n=115 (12-18 y); RCT: N=52 families (children 3-6 y)
Whiteside, 2016 [49]	Mayo Clinic Anxiety Coach	children and adolescents; anxiety symptom management	Mobile phone (IOS) app	Self-help; no equipment required	No	US $4.99; >169 (5-17 y; 2012 data)	US; English	Randomized, active comparator: n=10 (7-17 y)
Patwardhan, 2015 [50]	REACH	4th and 5th grade children (9-11 y); anxiety prevention and early intervention	Android platform	Not specified; no equipment required	No		US	Usability study: n=22 (9-11 years); Usability study: N=132+, 45 service providers
Vigerland, 2013 [51]; Vigerland, 2016 [52]	DARE program	8-12 y; treatment of anxiety	ICBT^c^; Web based	Minimal support therapist contact; combined parent-child intervention; no equipment required			Sweden; not specified	Pilot, uncontrolled: n=30 (8-12 y); RCT: n=93 (8-12 y) with 182 parents
Morgan, 2016 [53]; Morgan, 2017 [54]	Cool Little Kids	3-6 y; parents of anxious children	ICBT; Web based	Minimal support therapy; no equipment required	No		Australia; English	Randomized, uncontrolled: n=51 parents (children 3-6 y); RCT: N=433 parents (children 3-6 y)
Coyle, 2011 [55]	Pesky gNATs Island	9-17 y; treatment of anxiety	3-dimensional game CBT + mobile app (IOS and Android)	Professional; no equipment required	No	£150	UK and Ireland; English	Case studies: n=6 and n=15 (11-16 y); Professional survey (216 professionals working with adolescents)
Stallard, 2011 [56]	Think, Feel, Do	11-16 y; treatment of anxiety and depression	Computerized CBT-based CD-ROM	Minimal involvement from facilitators (non-CBT clinician): teachers, nurses, psychologist assistants, etc; no equipment required			UK; English	Pilot RCT: n=20 (11-16 y)
Cunningham, 2009 [57]; Wuthrich, 2012 [58]	Cool Teens Program	14-17 y; treatment of anxiety	Computerized CBT-based CD-ROM; Macromedia Flash MX (Windows and Mac)	Minimal therapist contact and phone; no equipment required			Australia; English	Pilot case series: 5 case studies (14-16 y); RCT: n=43 adolescents (14-17 y)
Calear, 2009 [59]	MoodGYM	Early intervention anxiety and depression	Web-based self-help CBT based	Teacher supervision	Answers to exercises and quizzes collected throughout the program	Free	Australia; English, Chinese, Finnish, Norwegian, Dutch	RCT: N=1477 (12-17 y)
Tillfors, 2011 [60]	Unnamed program for SAD^d^	Treatment of SAD	Internet-delivered CBT	Therapist	No		Sweden	RCT: N=19 (15-21 y)
Cox, 2010 [61]	Kids-accident website	Treatment of anxiety and posttraumatic stress disorder	Website; CBT based and resiliency theory	Parents (booklet)	No		Australia	RCT: N=85 children and adolescents (7-16 y)

^a^References [41,43,49] were found in clinicaltrials.gov and results have not yet been published.

^b^RCT: randomized controlled trial.

^c^ICBT: internet-delivered cognitive behavioral therapy.

^d^SAD: social anxiety disorder.

**Table 2:** Results from clinical trials: summary of published studies with efficacy data and anxiety diagnosis as primary outcome.

First author, year, reference	Program name	Sample	Study design	Diagnosis	% Primary diagnosis reduction at posttreatment^a^	% Primary diagnosis reduction at follow-up^a^
Khanna, 2010 [38]	CCAL^b^	N=49, 7-13 y, 67% male	RCT^c^: 3 arms (16 CCAL, 17 individual CBT^d^, 16 CESA^e^)	DSM-IV^f^SA^g^, SoP^h^, GAD^i^, SP^j^, PD^k^	At 12 weeks— CCAL: 81%, individual CBT: 70%, CESA: 19%	
Crawford, 2013 [40]	CCAL	N=17, 7-13 y, 71% male	Pilot: single arm	DSM-IV SA, SoP, GAD, SP	At 12 weeks— 87%	
Storch, 2015 [39]	CCAL	N=100, 7-13 y, 56% male	RCT: 3 arms (49 CCAL, 51 treatment as usual)	DSM-IV SA, SoP, GAD, SP, PD	At 12 weeks— CCAL: 55.1%, treatment as usual: 17.6%	
Spence, 2006 [45]	BRAVE	N=72, 7-14 y, 58% male	RCT: 3 arms (22 clinic, 27 clinic plus internet, 23 waitlist control)	DSM-IV SA, SoP, GAD, SP	At 10 weeks— clinic: 59.1%, clinic plus internet: 51.9%, waitlist control: 13%	At 6 months— clinic: 68.2%, clinic plus internet: 55.6%; At 12 months— clinic: 77.3%, clinic plus internet: 66.7%
March, 2009 [46]	BRAVE	N=73, 7-12 y, 45% male	RCT: 2 arms (40 ICBT^l^, 33 waitlist control)	DSM-IV SA, SoP, GAD, SP	At 10 weeks— ICBT: 30%, waitlist control: 10.3%	At 6 months— ICBT: 75%
Donovan, 2014 [47]	BRAVE	N=52, 3-6 y and parents, 46% male	RCT: 23 ICBT, 29 waitlist control	DSM-IV SA, SoP, GAD, SP	At 10 weeks— ICBT: 39.1%, waitlist control: 24.1%	At 6 months— ICBT: 52.2%
Spence, 2011 [48]	BRAVE	N=115, 12-18 y, 41% male	RCT: 3 arms (44 clinic, 44 ICBT, 27 waitlist control)	DSM-IV SA, SoP, GAD, SP	At 12 weeks— clinic: 29.5%, ICBT: 34.1%, waitlist control: 3.7%	At 6 months— clinic: 50.0%, ICBT: 54.5%; At 12 months— clinic: 68.2%, ICBT: 68.2%
Vigerland, 2013 [51]	DARE	N=30 (+57 parents), 8-12 y, 43% male	Single arm	DSM-IV SP	At 6 weeks— 33%	At 3 months— 47%
Vigerland, 2016 [52]	DARE	N=93 (+182 parents), 8-12 y, 45% male	RCT: 46 DARE, 47 waitlist control	DSM-IV SA, SoP, GAD, SP	At 10 weeks— DARE: 20%, waitlist control: 7%	At 3 months— DARE: 50%
Wuthrich, 2012 [58]	Cool Teens	N=43, 14-17 y, 37% male	RCT: 24 Cool Teens, 19 waitlist control	DSM-IV any anxiety disorder	At 12 weeks— Cool Teens: 41%, waitlist control: 0%	At 3 months— Cool Teens: 26%, waitlist control: 0%

^a^Proportions of children who were free of their primary anxiety diagnosis.

^b^CCAL: Camp Cope-A-Lot.

^c^RCT: randomized controlled trial.

^d^CBT: cognitive behavioral therapy.

^e^CESA: computer-assisted education, support, and attention control.

^f^DSM-IV: *Diagnostic and Statistical Manual of Mental Disorders* (Fourth Edition).

^g^SA: separation anxiety.

^h^SoP: social phobia.

^i^GAD: generalized anxiety disorder.

^j^SP: specific phobia.

^k^PD: panic disorder.

^l^ICBT: internet-delivered cognitive behavioral therapy.

#### Cognitive Behavioral Therapy–Based Programs for Children

We identified 7 programs for children aged 6 to 12 years old (see Table 1). Of the 7 programs, 3 were developed as mobile phone apps: SmartCAT [42], Mayo Clinic Anxiety Coach [62], and REACH [50].

Of the 3 apps, only REACH appeared to have a gaming component embedded, and was developed for prevention and early intervention purposes. The other 2 were built mainly to support the patients completing tasks and exercising the acquired skills in the real world. They provided psychoeducational contents, instructions, self-tests, and to-do lists. Anxiety Coach was a self-help app that focused on exposure exercises, while SmartCAT was used in a treatment program and included the app, the therapist portal, and a secure 2-way communication system, through which the therapist could monitor child activity, manage reward points, and send materials and messages to patients. To our knowledge, none of the mobile phone apps had published data derived from trials examining their efficacy. However, studies on the use of the apps, their utility, and users’ satisfaction yielded promising results [42,50,62]. The 4 computer-based programs Camp Cope-A-Lot [37-40] Treasure Hunt [44,63], BRAVE [45-48], and DARE [51,52] foresaw the involvement of therapist and parents at variable levels. BRAVE also had a self-help version.

The Camp Cope-A-Lot program presented a certain level of personalization, including elements that were customizable by the user to match their specific needs (eg, exposure tasks, speed at which they progress).

All but Treasure Hunt published efficacy data, with an effect in reducing anxiety diagnosis ranging from 20% to 80% (see Table 2 [38-40,45-48,51,52,58]). For Treasure Hunt, data from more than 200 children and 40 therapists indicated the program to be helpful in treatment, increasing child motivation, and strengthening the therapeutic relationship.

No significant differences were found in the controlled studies when comparing efficacy between computer-based and traditional face-to-face CBT approaches [38,39,45,46]. Interestingly, data from BRAVE showed that efficacy was maintained and even improved at follow-up (6 and 12 months), probably due to slower completion for the internet-based approach [45,46]. The relevance of the influence of time emerged also from the DARE studies [51,52].

#### Cognitive Behavioral Therapy–Based Programs for Adolescents

We identified 7 programs for the reduction of anxiety in adolescents, all based on CBT principles.

The BRAVE Web-based program had 1 version dedicated to children and 1 for adolescents (and their parents for each version). Graphics, sound, content, and examples that were used were appropriate for the developmental and cognitive levels of the 2 age ranges [48]. Another 2 programs were delivered via CD-ROM: Cool Teens [57] and Think, Feel, Do [56]. Think, Feel, Do was a software package developed to target both depression and anxiety symptoms in children and adolescents. Both programs foresaw therapist involvement and used a combination of multimedia types covering key CBT topics. Pesky gNATs Island [55] was a 3-dimensional computer game also based on CBT concepts developed to support traditional physical intervention through a therapist. It was coupled with a mobile app for iPhone and Android phones and tablets, available for free for patients playing the game with a therapist.

Results from efficacy and acceptability studies showed the computer-based programs to be acceptable to this age group and to have a favorable impact on clinical improvement [48,56,57,58].

Another 2 Web-based programs were a program (unnamed) addressing social anxiety [60] and the kids-accident website targeting posttraumatic anxiety [61]. The first one was based on a self-help manual with online feedback and targeted social anxiety only [60]. It appeared to mostly deliver content through text, with limited interaction and no gamification. The kids-accident website by Cox and colleagues [61] was based on CBT and resiliency theory. Data on efficacy of these programs showed preliminary evidence of effect. Finally, the MoodGYM program was tested in an adolescent school-based population to reduce symptoms of anxiety and depression [59], and showed some advantage—although not significant—of the intervention condition compared with the waitlist control on anxiety scores. MoodGYM is a Web-based, self-directed CBT program designed to prevent or decrease the symptoms of anxiety and depression in adolescents.

#### Cognitive Behavioral Therapy–Based Programs for Preschool Children

Several traditional programs for early intervention and prevention for young children were developed and showed preliminary evidence of their efficacy [64]. These focused on improving parent-preschool child interaction, by targeting the parent’s skills.

We located only 2 technology-based programs targeting the preschool age group. Cool Little Kids Online [53,54] was useful to parents for the acquisition of skills and strategies to help their child. Also, a modified version of the BRAVE Web-based program, with a parent-focused approach, was tested with preschool children and showed efficacy [47].

### Biofeedback-Based Programs

Biofeedback is a technique that teaches users to recognize and control their bodies’ functions, such as heart rate, respiration, muscle activity, and skin temperature, with the use of electronic instruments. It is commonly used for stress reduction, as it helps people control their stress response, by recognizing when they are stressed and employing relaxation techniques to reduce their physiological arousal [65].

We found 2 games specifically designed to address anxiety in children using biofeedback: Relax to Win [66] and Dojo [67,68]. In these games, players acquired relaxation techniques, such as deep breathing and progressive muscle relaxation, and practiced them. Generally, the games visually reproduced challenging or stressful situations that the player could overcome through control of their own physiological and emotional conditions; they progressed successfully through the game if they are able to keep calm. A device captured skin conductivity and transferred data to the mobile phone, triggering real-time feedback. The studies conducted on Relax to Win and Dojo did not allow reaching conclusions regarding their effectiveness, and further studies are needed to assess the ability of this approach to reduce stress in children and to contribute to the management of anxiety.

Another program incorporated several strategies into a game aimed at school-aged children (8-12 years) with anxiety: Mindlight used neurofeedback, exposure training, attention bias modification, relaxation, and mindfulness techniques. Data from a randomized controlled study showed promising, although not conclusive, efficacy results [69,70]. One also indicated that Mindlight was as effective as traditional CBT in the prevention of anxiety [70].

Table 3 [66-70] summarizes the biofeedback-based programs.

### Other Approaches

Other computer-based programs based on theoretical approaches other than CBT, such as mindfulness, have been studied (see Table 4 [71-73]).With respect to mindfulness, the clinical application of and research on mindfulness-based interventions has been growing in the last decades, and as a recent meta-analysis of Web-based mindfulness-based interventions for improving mental health including anxiety showed, data indicate their effectiveness in reducing anxiety and depression symptom severity, with effect sizes between 0.3 and 0.8 [6]. However, this meta-analysis focused on adults and not on youth. Even though several reviews and articles on mindfulness-based apps are available that may target children [74], we found no published results of randomized controlled trials on their effectiveness or on their usability and acceptability.

A Web-based self-help program [71] based on problem-solving therapy was developed for the treatment of depression and anxiety symptoms. It simply provided content through text and provided feedback from the clinician through email. It did not yield evidence of efficacy.

Finally, we identified a therapeutic 3-dimensional game for adolescents, based on solution-focused therapy, called Personal Investigator [72,73]. Solution-focused therapy is a goal-oriented form of therapy based on the assumption that individuals have some knowledge of what they would improve in their life and have the skills necessary to develop solutions. It focuses more on the present rather than the past and on creating future solutions than on analyzing problems. In this game, the teenager played the role of a personal investigator hunting for solutions to personal problems. The game was used during sessions with therapists. Data suggested that the game may favor engagement, high motivation, and enjoyment of the user and a rapid development in the therapeutic relationship [72,73].

**Table 3:** Biofeedback-based serious games, for children and adolescents: summary of features of technology-based tools using biofeedback.

First author, year, reference	Tool name	Target population	Theoretical framework and platform	External support	Automated decision support and data collection	Country and language	Structured pilot or trial
Sharry, 2003 [66]	Relax To Win	Children, relaxation training	Biofeedback, computer video game (3-dimensional); 5 sessions	Professional; required equipment: electrocardiogram, electroencephalogram	Yes, biofeedback	English	1 case study (12-year-old boy)
Scholten, 2016 [67]; Schuurmans, 2015 [68]	Dojo	11-15 y; anxiety reduction	Biofeedback, 3-dimensional game	Equipment: biofeedback hardware IOM (Wild Divine)	Yes, biofeedback	Netherlands	Pilot uncontrolled: N=8 (mean 14.38, SD 1.6 y); RCT^a^: N=138 (11-15 y)
Schoneveld, 2016 [69]; Schoneveld, 2018 [70]	Mindlight	8-16 (children and adolescents); anxiety symptoms	3-dimensional serious computer game; neurofeedback, CBT^b^-based exposure training, attention bias modification	Professional; Equipment: Neurosky neurofeedback headset		Netherlands; Dutch	RCT: n=136 (7-13 y); RCT: N=120 (8-16 y); RCT noninferiority: N=174 (7-12 y)

^a^RCT: randomized controlled trial.

^b^CBT: cognitive behavioral therapy.

**Table 4:** Other approaches: summary of features of technology-based tools using other approaches.

First author, year, reference	Tool name	Target population	Theoretical framework and platform	External support	Country and language	Structured pilot or trial
Coyle, 2009 [72]; Coyle, 2005 [73]	Personal Investigator	Treatment of anxiety and depression	3-dimensional game, solution-focused therapy; Atmosphere JavaScript application programming interface, Macromedia Flash MX	Professional; No equipment required	Ireland; English	Pilot uncontrolled: n=4 (13-16 y); n=22 (10-16 y)
Hoek, 2012 [71]	Internet-based problem-solving therapy (unnamed)	Adolescents; anxiety and depression	Web based	Guided self-help, email feedback from clinician	Netherlands; Dutch	Randomized controlled trial: N=45 (12-21 y)

As a concluding finding, it is important to note that an issue that was emphasized in the case of new technology-based interventions, regardless of the approach that was used or the age range for which the intervention was designed, is related to acceptability and adherence. Dropout is high even in traditional therapy and is generally higher in self-help programs [75].

Although all the programs had positive data on acceptability and user satisfaction, data arising from the studies indicate that adherence should receive some attention. For example, data from BRAVE showed that a large percentage of users did not complete the program in the expected time (at a pace of 1 session per week).

## Discussion

### Principal Findings

Research demonstrates promise for the use of computer technology in the treatment of adult anxiety [76-78]. A smaller volume of data from fewer studies on the treatment of childhood anxiety is available but is promising [79]. A meta-analysis demonstrated that internet-based interventions are effective in reducing anxiety symptom severity in youth compared with no intervention, and their effect may be comparable with that of face-to-face interventions [80]. However, considering the large number of Web-based programs that can be found on the internet and apps that can be downloaded on mobile phones and tablets, only a minority have been systematically tested and have published data on feasibility, acceptability, efficacy, and effectiveness.

We identified 19 technology-based programs that are available for children and adolescents with anxiety. Much less effort has been dedicated to the development of programs targeting preschool children, and the only ones we could locate (Cool Little Kids Online and a BRAVE version for preschool children) focused on improving parental skills as a way to indirectly help the preschoolers. One possible explanation for this is that the developmental stage of preschool children constitutes a challenge for the application of commonly used CBT programs, which is the evidence-based approach that has been most frequently translated into technology-based interventions [81]. It is important to note that this finding is in agreement with a recent review that evaluated the use of CBT-informed behavioral intervention technologies for the prevention and treatment of depression and anxiety among youth [30], which found that the child population of 5- to 12-year-olds received less attention than children in the age range of 12-17 years. This reveals a gap of addressing the needs of children younger than 8 years for prevention purposes, before instances of stress or subclinical anxiety escalate to clinical anxiety. Therefore, alternative approaches, based on developmentally appropriate games targeting preschoolers, should be developed and examined to determine their potential efficacy in early prevention.

Given the high prevalence of anxiety symptoms and disorders in youth, their negative impact on child development and performance, and the increased risk of developing related mental disorders in later ages, prevention, and early intervention, are very important. Furthermore, the median age of onset has been reported to be 6 years [3]. Thus, much more effort should be put into the development of programs targeting preschool children. Intellectual, language, and socioemotional developmental domains of preschool children present a particular challenge for an eHealth or mHealth program, and joint parent-child programs need to be developed and evaluated for their efficacy.

In a world where computers, the internet, and mobile devices such as smartphones and tablets are widely used, computer and mobile technology offers a novel format for the delivery of treatment for child anxiety, which offers a reduction in costs, increased accessibility, and potentially standardization of content and delivery [82] and avoidance of stigma [83].

Furthermore, personal computing-based interventions have the potential to be delivered to a very large number of people compared with traditional face-to-face interventions. Data indicate that the average 8- to 14-year-old spends more than 1 hour per day playing digital games [80] and, by the time adolescents reach the age of 21 years, they will have spent at least 10,000 hours playing these games [84]. As of January 2014, 58% of the US population owned a smartphone, and it is predicted that by 2020, 90% of the world’s population over the age of 6 years will have a mobile phone [85]. Furthermore, recent data indicate that 45% of US adults own a tablet; this percentage has substantially increased since the Pew Research Center began measuring tablet ownership in 2010, when only 4% of adults in the United States owned a tablet [86].

These data indicate that smartphones and mHealth programs hold great promise for widespread prevention. Despite this evidence, little has been done so far in this area: among all the programs (n=19) that we identified, only 5 used tablets or smartphone technology, of which only 2 (1 CBT-based and 1 biofeedback app) combine mobile technology and gaming.

The vast majority of the programs we evaluated had the aim of treating children or adolescents with anxiety disorders, and almost all were designed to support traditional therapy or foresee the intervention of a therapist. To our knowledge, among the CBT-based programs, only REACH was designed for prevention and early intervention, and only the Mayo Clinic Anxiety Coach was created as a self-help program; BRAVE also had a self-help version. Among the other 5 non–CBT-based programs, 2 (Relax to Win and Personal Investigator) foresaw their use within a traditional treatment program.

An issue that has been emphasized in the case of new technology-based interventions is related to acceptability and adherence. Dropout is high in self-help programs [75], and higher than in traditional therapy. Although all the programs had positive data on acceptability and user satisfaction, the same attention was not given to adherence issues. This gives rise to two considerations. The first consideration is that the pace at which users of a technology-based intervention move forward may be different from traditional face-to-face interventions and may vary depending on characteristics of the program or characteristics of the users. Data showed that it may take anywhere from 18 days to 254 days for people to form a new habit, depending on the behavior, the person, and the circumstances [87]. The second consideration is that, although games are intrinsically motivating by offering fun and rewards to children, persuasive design elements that maximize adherence and personalization should be considered when developing a game. For example, the developers of REACH used a user-centric approach, with iterative feedback, participatory design, and end-user validation.

Data to understand use of and adherence to the eHealth or mHealth program are needed. Volume, quality, and type of data vary widely across programs. To our knowledge, automated data collection is very limited. Treasure Hunt (XML) and SmartCAT (Web interface) used automated data collection and provided that information to the therapist. Anxiety Coach collected data concerning the download and use of the app. Also for biofeedback-based programs, our understanding is that data were collected for real-time feedback and progression of the game, but were not collected and analyzed for the purpose of understanding the correlation between the use of the program and long-term efficacy of an intervention. Although this raises the problem of privacy and data security, automated data collection has the potential to also assess effectiveness of the programs on a large scale.

Of the 19 programs that we evaluated, 15 reported efficacy data, and randomized controlled trials were performed for all but 1 of them. All CBT-based programs showed positive effects in reducing anxiety, and no difference was noted when those were compared with traditional intervention.

Biofeedback-based programs also showed some evidence of effect in reducing stress. These games, however, addressed only the biophysiological component of anxiety. Relaxation training can be particularly effective in addressing the physiological arousal of anxiety; however, data from the technology-based biofeedback games are yet to conclusively show their efficacy.

The 2 available studies on programs based on other approaches did not demonstrate efficacy. Reasons for not showing efficacy may reside in low sample power, in the weakness of a theoretical approach, or in the program format itself. Further studies are needed to clarify the usefulness of these approaches.

eHealth and mHealth is a rapidly growing field, and several programs have been developed to support the treatment of diseases including psychiatric disorders. In particular, this approach has been shown to be effective in adults with anxiety. As a result, several programs for children have been created in the last decades. Available data indicate this is a promising approach to enhance treatment and make it accessible to a larger percentage of children in need. However, the field is still in its infancy and requires the development of self-help programs in order to be suited for wide distribution for prevention purposes. Conventional prevention approaches are unable to tailor interventions to the diverse needs and learning paces of at-risk children. eHealth and mHealth programs have this potential: by design, digital games are fun, engaging, and able to elicit powerful emotions; can dynamically adjust the degree of difficulty and reinforce the player’s actions; and can be used in natural settings and everyday life, at the pace and needs of the users. Furthermore, they may support learning by eliciting positive emotions [88] and by using a more experiential approach to convey CBT concepts and skills.

As a concluding remark, even though at least 120 apps targeting child anxiety are accessible in widely used marketplaces, namely Google Play for Android and Apple’s App Store for iOS, only roughly half of them include at least one evidence-based approach [89]. It is unclear whether the evaluated apps have peer-reviewed publications to support their acceptability, usability, or effectiveness. There is, therefore, a research-to-practice gap that limits the availability of evidence-based treatments for youth anxiety and there are valid concerns about the quality of readily accessible apps for youth anxiety [89].

### Challenges

Based on the findings of this review and a previous study [68], this field has several challenges to overcome: privacy and data security; automated data collection for assessing use and effectiveness; users’ input by design to maximize acceptability, adaptability to personal differences, and engagement; development of self-help prevention programs; wide dissemination of such programs and special attention given to repurposing to address cultural differences (eg, translation into languages other than English and adapting to local needs and expectations; considering specific difficulties related to minority groups, migrants, and social integration; involvement of government and public health agencies); keeping up with the fast progress of technology; filling the gap for preschool children; and funding to support development and sustainability (ie, long-term plans for software updates; effectiveness studies with large sample size, and long-term longitudinal follow-up).

### Conclusions

Smart device use is ubiquitous among children; however, research and development of stress management interventions is not fully taking advantage of new technologies. Data indicate that this is a promising approach to enhance treatment and make it accessible to a larger percentage of children in need. Most interventions, including CBT-based programs and biofeedback approaches, require the presence of human professionals and biosensors, respectively, and are not easily deployable at the population level. There is a clear need and a broad potential for the development of self-help programs, to be suited for a wide distribution, and for prevention purposes, especially at younger ages.

## Appendix

Multimedia Appendix 1 Glossary.

## Abbreviations

CBT: cognitive behavioral therapy
CCAL: Camp Cope-A-Lot
CESA: computer-assisted education, support, and attention control
DSM-IV: Diagnostic and Statistical Manual of Mental Disorders (Fourth Edition)
GAD: generalized anxiety disorder
ICBT: internet-delivered cognitive behavioral therapy
PD: panic disorder
RCT: randomized controlled trial
SA: separation anxiety
SoP: social phobia
SP: specific phobia
