# Supplementary material for: eHealth Interventions for Anxiety Management Targeting Young Children and Adolescents: Exploratory Review
Source: JMIR Pediatr Parent. 2018 May 10;1(1):e5. doi: 10.2196/pediatrics.7248 (PMC6716078; doi:10.2196/pediatrics.7248)
Supplement: Multimedia Appendix 1 [file pediatrics_v1i1e5_app1.pdf]

GLOSSARY

*Acceptability***:** Indicates to what extent a new idea, program, process, or measure is judged as suitable, satisfying, or attractive to program recipients. It includes the evaluation of

- Satisfaction
- Intent to continue use
- Perceived appropriateness

Adherence to (or compliance with) a therapeutic regimen is generally defined as the extent to which patients follow recommendations of the health care provider: take medications, follow diets, and execute life style changes as prescribed.

Effectiveness trials (pragmatic trials) measure the degree of beneficial effect under "real-world" clinical settings. Hence, hypotheses and study designs of an effectiveness trial are formulated based on conditions of routine clinical practice and on outcomes essential for clinical decisions.

Efficacy trials determine whether an intervention produces the expected result under ideal circumstances. (See Gartlehner et al [1] for distinction between efficacy and effectiveness.)

Feasibility: Refers to the viability of an idea, and consists of an analysis and evaluation of a proposed project to determine if it is feasible technically, within the estimated cost, and with profitability. The evaluation of feasibility includes a variety of aspects of intervention delivery: demand, implementation, practicality. In addition, it incorporates acceptability.
